# Venous Thromboembolism after Allogeneic Pediatric Hematopoietic Stem Cell Transplantation: A Single-Center Study

**DOI:** 10.4274/tjh.2013.0066

**Published:** 2015-08-01

**Authors:** Fatih Azık, Dilek Gürlek Gökçebay, Betül Tavil, Pamir Işık, Bahattin Tunç, Duygu Uçkan

**Affiliations:** 1 Ankara Children’s Hematology Oncology Hospital, Clinic of Pediatric Hematology Oncology, Ankara, Turkey

**Keywords:** Thrombosis, Pediatric stem cell transplantation, Prothrombotic risk factors

## Abstract

**Objective:**

Venous thromboembolism (VTE) in children who undergo hematopoietic stem cell transplantation (HSCT) has high morbidity. The aim of this study is to assess the incidence of VTE in allogeneic pediatric HSCT recipients and the contribution of pretransplant prothrombotic risk factors to thrombosis.

**Materials and Methods:**

We retrospectively evaluated 92 patients between April 2010 and November 2012 undergoing allogeneic HSCT who had completed 100 days post-HSCT. Before HSCT, coagulation profiles; acquired and inherited prothrombotic risk factors including FV G1691A (factor V Leiden), prothrombin G20210A, methylenetetrahydrofolate reductase (MTHFR) C677T, and MTHFR A1298C mutations; and serum homocysteine and lipoprotein (a), plasma antithrombin III, protein C, and protein S levels were obtained from all patients.

**Results:**

In the screening of thrombophilia, 8 patients (9%) were heterozygous for factor V Leiden, 5 (6%) were homozygous for MTHFR 677TT, 12 (14%) were homozygous for MTHFR 1298CC, and 2 (2%) were heterozygous for prothrombin G20210A mutation. We observed VTE in 5 patients (5.4%); a prothrombotic risk factor was found in 3 out of these 5 patients, while 4 out of 5 patients had central venous catheters. It was determined there was no significant relationship between VTE and inherited prothrombotic risk factors.

**Conclusion:**

VTE after HSCT seems to be a low-frequency event that may be due to low-dose, low-molecular-weight heparin prophylaxis, and the role of inherited prothrombotic risk factors cannot be entirely excluded without a prospective study.

## Abstract (tr)

**Amaç:**

Hematopoetik kök hücre nakli (HSCT) yapılan hastalarda venöz tromboembolizm (VTE) yüksek morbiditeye sahiptir. Bu çalışmanın amacı pediatrik allojenik HSCT alıcılarındaki VTE insidansını belirlemek ve pretransplant protrombotik risk faktörlerinin trombozdaki rolünü saptamaktır.

**Gereç ve Yöntemler:**

Nisan 2010-Kasım 2012 tarihleri arasında allojenik HSCT yapılan ve HSCT sonrası 100 gününü tamamlayan 92 hasta retrospektif olarak değerlendirildi. Kök hücre nakli öncesinde tüm hastalardan koagülasyon profili, kazanılmış ve konjenital protrombotik risk faktörlerinden faktör V Leiden, protrombin G20210A, metilentetrahidrofolat redüktaz (MTHFR) C677T, MTHFR A1298C mutasyonları ile serum homosistein ve lipoprotein (a), plazma antitrombin III, protein C ve protein S düzeyleri alındı.

**Bulgular:**

Trombofili taramasında; toplam sekiz hasta (%9) faktör V Leiden heterozigot, beş hasta (%6) MTHFR 677TT homozigot, 12 hasta (%14) MTHFR 1298CC homozigot ve iki (%2) hasta prothrombin G20210A heterozigot mutasyonuna sahipti. Venöz tromboembolizm beş hastada (%5,4) izlendi; herhangi bir protrombotik risk faktörü beş hastadan üçünde tespit edilirken, dört hastanın santral venöz kateteri vardı. Venöz tromboembolizm ile kalıtsal protrombotik risk faktörleri arasında anlamlı ilişki saptanmadı.

**Sonuç:**

Hematopoetik kök hücre nakli sonrasında VTE; muhtemelen düşük dozda düşük moleküler ağırlıklı heparin kullanımına bağlı olarak az sıklıkta görülebilen bir komplikasyon olup, prospektif bir çalışma yapılmaksızın kalıtsal protrombotik risk faktörlerinin trombozdaki rolünün tam olarak dışlanması mümkün değildir.

## INTRODUCTION

Thrombotic events are common complications in children receiving hematopoietic stem cell transplantation (HSCT). It is suggested that endothelial damage and activation of coagulation induced by pretransplant conditioning regimens may contribute to the development of thrombotic events [1]. Previous studies evaluating coagulation profiles of patients undergoing HSCT revealed altered hemostatic parameters and increased thrombin generation during HSCT. Additionally, activation of coagulation, acquired anticoagulant deficiencies, and decreased fibrinolysis were noted in HSCT patients with microangiopathic disease [2,3]. In a pediatric study, a persistent state of coagulation was considered to cause the increase in plasma levels of thrombin-antithrombin complexes and decreased levels of plasma antithrombin III (ATIII) and protein C in the early HSCT period [4]. In addition, children receiving HSCT require long-term central venous catheters (CVCs) for administering medications, blood products, and parenteral nutrition and for daily blood sampling [5]. CVCs and inherited prothrombotic risk factors may also contribute to thrombotic complications in HSCT recipients. In the English-language literature, there are contradictory studies regarding the effect of inherited prothrombotic risk factors on thromboembolism in children undergoing HSCT [6]. In this study we evaluated the incidence of venous thromboembolism (VTE) in allogeneic pediatric HSCT recipients and the contribution of pretransplant prothrombotic risk factors to thrombosis.

## MATERIALS AND METHODS

One hundred consecutive patients underwent HSCT from April 2010 to November 2012, and 92 of these transplants were allogeneic; these cases were enrolled in the study. All patients completed 100 days post-HSCT and data were collected retrospectively. All transplants were from related donors. Patients were admitted for various hematologic/oncologic diseases or immune deficiency disorders. All of the patients had CVCs and they were flushed, and the dressing was changed twice weekly. Catheters remained in place until hematopoietic recovery in all patients unless the catheter was proven to be infected, had mechanical complications, or was occluded with thrombus.

Before insertion of CVCs, blood samples were obtained from all patients for coagulation parameters (prothrombin time, activated partial thromboplastin time, fibrinogen, d-dimer) and inherited thrombophilic factors including factor V G1691A (factor V Leiden), prothrombin G20210A, methylenetetrahydrofolate reductase (MTHFR) C677T, and MTHFR A1298C mutations and serum homocysteine, lipoprotein (a), plasma ATIII, protein C, and protein S levels. ATIII, protein C, and protein S levels were referenced for age-specific values [7]. Cut-off values were 30 mg/dL for serum lipoprotein (a) and 12 µmol/L for serum homocysteine [8]. Patients with thrombosis were checked for family history of thrombosis. None of the patients had past history of thrombosis prior to HSCT. Written informed consent was obtained from all patients’ parents.

All patients stayed in HEPA-filtered isolation rooms and received standard regimens for the prevention of infectious complications (acyclovir for herpes simplex and varicella zoster, co-trimoxazole for Pneumocystis jirovecii, metronidazole for anaerobic infections, ciprofloxacin for bacterial infections, and fluconazole for Candida prophylaxis). All cellular blood products were leukocyte-depleted and irradiated prior to transfusion. While most of the HSCT recipients received cyclosporine and methotrexate for prevention of graft-versus-host disease (GVHD), a few of them with Fanconi aplastic anemia received cyclosporine and steroids. All patients received low-molecular-weight heparin (LMWH) enoxaparin at a dose of 0.8 mg/kg/day by subcutaneous injection for prevention of hepatic veno-occlusive disease (VOD) for 28 days. If the patient was bleeding or developed refractory thrombocytopenia, the LMWH dose was withheld. When VOD was diagnosed according to the Seattle criteria [6], defibrotide therapy was started at a dose of 25 mg/kg/day and LMWH was stopped. Myeloid engraftment was defined as the first of 3 consecutive days when the neutrophil count was >0.5x109/L and platelet engraftment as the first of 7 consecutive days with an unsupported platelet count of >20x109/L. Echocardiography was performed in all patients before the conditioning regimen and 1 month after HSCT or before discharge. When a patient had clinically suspected thrombosis, Doppler ultrasonography (US), echocardiography, and, if required, magnetic resonance (MR) imaging and angiography were performed.

### Statistical Analysis

Analysis of data was primarily descriptive, using standard deviations, ranges, and mean and median values. Categorical variables were analyzed using the chi-square test and continuous variables were analyzed using Student’s t test. All analyses were performed using SPSS 18 for Windows (SPSS Inc., Chicago, IL, USA). When p≤0.05, it was considered statistically significant.

## RESULTS

A total of 92 patients (57% males and 43% females) received allogeneic HSCT; median age was 8.5 years (range: 0.5-16 years). Forty-one (44.6%) patients had malignant, 39 (42.4%) had nonmalignant, and 12 (13%) had immunodeficiency disorders. The patients and transplant characteristics are summarized in Table 1. Hickman double lumen catheters were inserted in 88 (95%) patients and 20 (22%) of them had had port catheters previously. Hickman catheters could not be inserted in 4 (5%) of the patients because of repeated CVC insertions or radiation therapy scars; therefore they had nontunneled jugular or femoral catheters during HSCT. Twenty-two of the central lines were removed during the study period as 16 of them became infected, 4 had mechanical complications (3 ruptures, 1 malposition), and 2 were blocked. Overall, 5.4% (5 out of 92) of the patients developed VTE. One patient had cerebral sinovenous thrombosis, 1 had portal vein thrombosis, and 3 had CVC-related thrombosis (2 at the right atrium and 1 at the femoral vein). Prothrombotic risk factors were found in 3 of these 5 patients, while 4 out of 5 patients had CVCs. One of the patients who had thrombosis was heterozygous for the MTHFR C677T polymorphism and had increased serum lipoprotein (a), 1 was heterozygous for the MTHFR A1298C polymorphism, and 1 had hyperhomocysteinemia without any mutation. In 2 other patients who had thrombosis, prothrombotic risk factors could not be detected. There was no statistically significant difference between HSCT recipients who had or did not have thrombosis with regard to prothrombotic risk factors (p>0.05).

There was no previous history of VTE in first-degree relatives in any of the patients who developed VTE undergoing HSCT. None of the patients had homozygous factor V Leiden or homozygous prothrombin G20210A mutation, or deficiency of protein C, protein S, or ATIII. In total, 8 (9%) of the patients were heterozygous for the factor V Leiden mutation and none of them had thrombosis. Two (2%) patients were heterozygous for the prothrombin G20210A mutation, 5 patients (6%) were homozygous for MTHFR 677TT, 12 (14%) were homozygous for MTHFR 1298CC, and no thrombotic event occurred. Prothrombotic risk factors of the HSCT recipients are summarized in Table 2.

Three of 5 patients with VTE diagnosis had immunodeficiency disorders whereas the other 2 patients had acute lymphoblastic leukemia. All of the CVC-related thrombosis occurred in a subgroup of 15 patients (27%) who developed acute GVHD of any grade following HSCT. There was no association found between patients with or without thrombosis with regard to malignant or nonmalignant diseases, stem cell sources, or myeloablative or nonmyeloablative preparative regimens. Sixteen of the 92 patients (17%) had VOD; 2 of them had mild VOD according to the Seattle criteria and received supportive treatment, and 14 of them had moderate VOD and received defibrotide therapy [6]. The median time to VTE following HSCT was 58 days (range: 25-66 days). All patients were treated with LMWH at a dose of 1 mg/kg subcutaneously twice a day. No severe bleeding complications occurred during anticoagulant therapy.

The patient with cerebral sinovenous thrombosis was admitted to the hospital complaining of headache and nausea at day +66 after HSCT. She was receiving steroid therapy for acute GVHD. Papilledema was noticed on her physical examination. Although her cranial computed tomography and MR imaging results were normal, lumbar puncture showed increased intracranial pressure (>300 mmH2O). MR venography revealed marked paucity of flow in the left transverse and sigmoid sinuses. She was treated with LMWH for 6 months, and then no obstruction was shown on MR venography and she received a prophylactic dose of acetylsalicylic acid for 6 months. The 2 patients who had right atrial thrombosis were asymptomatic; their CVCs were obstructed and thrombi were seen on echocardiography. They were treated with LMWH until CVC removal and echocardiography showed no thrombus in the right atrium. Swelling of the right leg was noticed after removal of the catheter in the patient with femoral vein thrombosis, and Doppler US showed obstruction in the femoral vein. He was treated with LMWH for 6 months and thrombosis dissolved in the second month, as shown on Doppler US. The patient with portal vein thrombosis had a second HSCT because of graft failure. Despite no inherited prothrombotic risk factor, he had pancreatitis and moderate VOD, and so he received defibrotide therapy and enoxaparin was stopped. Because he had hyperbilirubinemia and hepatomegaly, Doppler US was performed and an obstruction was seen in the right branch of the portal vein. He was treated with LMWH for 1 month and thrombosis dissolved within 2 weeks, as shown on Doppler US. Thereafter, he died from grade IV GVHD with gastrointestinal tract and skin involvement. Characteristics of patients with thrombosis are summarized in Table 3.

## DISCUSSION

Prior studies have suggested that thrombosis occurs in 4.4%-9% of pediatric HSCT patients, and the impacts of inherited prothrombotic risk factors remain controversial [4,9,10]. In the present study, 5 out of the 92 patients (5.4%) had VTE; prothrombotic risk factors was found in 3 patients with thrombosis, while 4 had CVCs. CVCs have improved the quality of care in children with hematological/oncological diseases or undergoing HSCT, and thrombosis is one of their most frequent secondary complications [11]. The incidence of catheter-related thrombosis in children with underlying hematological or malignant disorders ranges from 4% to 50% [12,13,14]. Catheter-related thrombosis occurred in 4 out of 92 patients (4.3%) receiving HSCT in our study. Knöfler et al. evaluated inherited thrombophilia and CVC-related thrombosis with malignancy; an inherited prothrombotic risk factor was determined in 23% (17 out of 77) of the subjects and in 64% (7 out of 11) of the subjects with thrombosis. They concluded that patients with combined inherited prothrombotic risk factors were particularly at risk for thrombotic events [15]. Revel-Vilk et al. evaluated 262 patients with malignancy and there was no association found between thrombophilia and CVC-related thrombosis, but a positive family history of thrombosis was related to thrombotic episodes [16]. Similarly, in the present study, we found no difference between HSCT recipients who had or did not have VTE with regard to prothrombotic risk factors. On the contrary, Abdelkefi et al. studied 171 HSCT recipients and CVC-related thrombosis was found in 7.6% of subjects; those who had at least 1 of the inherited prothrombotic markers had a 3.3-fold increased risk of thrombosis. In addition, they found that absence of heparin prophylaxis was also associated with CVC-related thrombosis [17]. Though there is some evidence that a prophylactic continuous infusion of heparin can prevent thrombosis in patients with CVC, Lagro et al. showed that there was no effect of using prophylactic nadroparin (a LMWH) on the incidence of CVC-related thrombosis in HSCT recipients [18,19]. We used enoxaparin at a dose of 0.8 mg/kg/day for prevention from VOD for 28 days, which may have offered some protection against the development of VTE.

We observed acute GVHD in 3 of 4 patients with VTE. Vascular and endothelial injury that occurs as a consequence of GVHD may contribute to inflammation, which increases the risk of VTE in allogeneic transplant recipients. Additionally, the use of immunosuppressive treatment for acute GVHD has not been fully investigated in regard to the risk of developing VTE [20].

In the present study, VTE was observed in 5.4% of pediatric HSCT recipients, which is in accordance with previous reports. We found no relationship between thrombosis and prothrombotic risk factors. We concluded that VTE seems to be a low-frequency event, which may be due to low-dose LMWH prophylaxis, and the role of inherited prothrombotic risk factors cannot be entirely excluded without a prospective study. Application of low-dose LMWH has been useful for preventing VTE after HSCT. Since all patients in our study had CVCs, the role of CVCs on thrombosis cannot be determined and acquired hemostatic alterations after HSCT are still speculative in VTE. In order to decrease CVC-related complications after HSCT, CVCs should be removed as soon as possible after the expected work is done.

## Figures and Tables

**Table 1:**
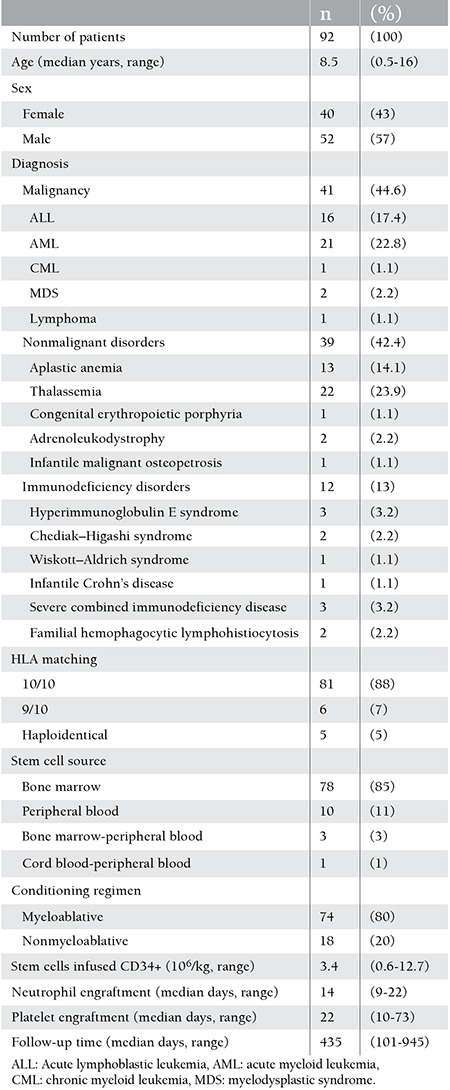
Subject demographics and the characteristics of the stem cell transplants.

**Table 2:**
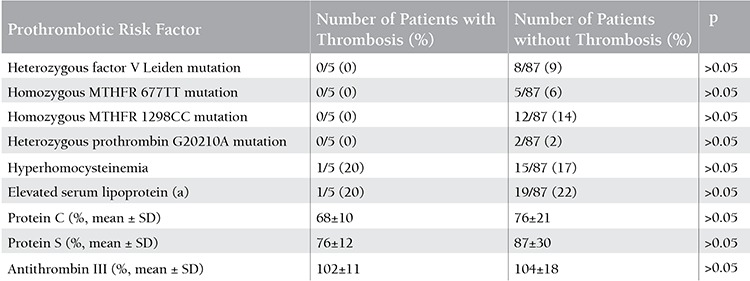
Characteristics of hematopoietic stem cell transplantation recipients with and without prothrombotic risk factors.

**Table 3:**
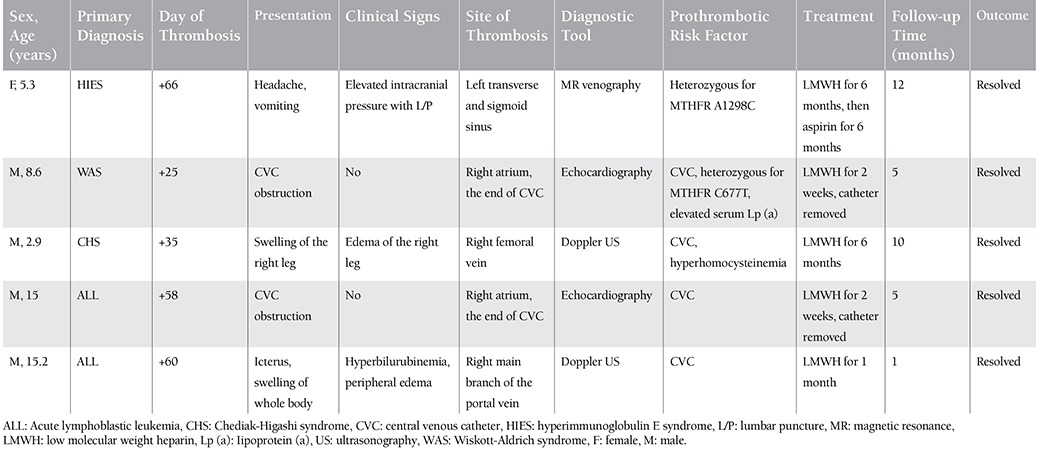
Clinical and laboratory findings of patients with thrombosis.
